# Antiobesity and antidyslipidemic properties of Clitoria ternatea petals aqueous extract against rats induced by high-fat diet

**DOI:** 10.55730/1300-0144.5805

**Published:** 2023-12-18

**Authors:** I Gede Aswin Parisya SASMANA, Desak Made WIHANDANI, I Gede Krisna Arim SADEVA, Wilson HALIM, Putu Putri AGUSTINI, Lalita SAMALA, I Gusti Ngurah Agung Surya PRATAMA, Lidiya Nuraliza RACHMAWATI, Aizar Vesa PRASETYO, Ni Komang Ayu Amanda Setiari JAYA

**Affiliations:** 1 Undergraduate Student, Faculty of Medicine, Udayana University, Denpasar, Indonesia; 2 Department of Biochemistry, Faculty of Medicine, Udayana University, Denpasar, Indonesia

**Keywords:** Anthocyanin, *Clitoria ternatea*, dyslipidemia, high-fat diet, in vivo, obesity

## Abstract

**Background/aim:**

Obesity is a chronic metabolic disease involving dysregulation of fat metabolism that affects 13% of the world’s population. Obesity has been linked to dyslipidemia with a lot of complication, including stroke, chronic kidney disease, fatty liver disease, and so on. One of the natural resources that have several potential effects including anticholesterol, antiobesity, and antidyslipidemia is the butterfly pea (*Clitoria ternatea*/CT). CT’s petal has been found to contain high levels of anthocyanins and tannins that can inhibit the biosynthesis of cholesterol and lipid. This study aims to investigate the antiobesity and antidyslipidemic effects of *Clitoria ternatea* extract (CTE).

**Materials and methods:**

The CTE was obtained through the aqueous extract method and then was investigated using spectrophotometry to determine anthocyanin and tannin content. The effect of CTE against a high-fat diet (HFD)-induced rat model was measured by weight and obesity index, lipid profile (total cholesterol (TC), triglycerides (TG), and HDL-C), and histopathology analysis.

**Results:**

CTE showed total anthocyanin and tannin content of 78.0943 mg/100 g and 1424.90 mg/100 g, respectively. The data analysis also showed significantly different within groups (p < 0,05), especially between HFD and HFD + CT750 groups on the cholesterol (MD 111.12 mg/dL; 95% CI (99.57 to 122.67); p < 0.001), LDL (MD; 76.38 mg/dL; 95% CI (56.77 to 96.00); p < 0.001), VLDL (MD 0.37 mg/dL; 95% CI (0.18 to 0.57); p < 0.001), body weight (MD: 56.20 g; 95% CI (13.89 to 98.51); p = 0.012); and thickening of tunica layer in the thoracic aorta (MD 22.76 μm; 95% CI (20.11 to 24.4); p < 0.001).

**Conclusion:**

This study shows that *Clitoria ternatea* petals aqueous extract promotes amelioration of the lipid profile, body weight, and tunica thickness in rats with the high-fat diet.

## 1. Introduction

High-fat diets and sedentary lifestyles keep rising in the general population, resulting in an imbalance between calorie intake and energy expenditure. High fat diet consumption increase up to 35.6% from 1991 and sedentary lifestyles prevalence increase up to 20% from 2000 that lead to several metabolic diseases, including obesity and dyslipidemia [1,2]. Obesity is a chronic disorder of fat metabolism characterized by excessive accumulation of fat in the body that can be a risk factor for several diseases, including dyslipidemia, diabetes, acute coronary disease, hypertension, and others [3]. Currently, obesity has become an epidemic in the United States, with the number of cases increasing every year. Based on data from the World Health Organization (WHO), obesity affected 13% of the world’s population in 2016 [4]. Obesity can stimulate oxidative stress through numerous mechanisms and lead to the increase of free radical levels and the decrease of antioxidant activity levels, resulting in a chronic systemic inflammatory condition in the body that leads to systemic organ disruptions [5].

Obesity is closely related to dyslipidemia, an imbalance of the lipid profile in the body that is found in about 60%–70% of patients with obesity [6]. Approximately, dyslipidemia contributes to 29.7% of disability-adjusted life years (DALYs) and 2.6 million deaths globally [7]. This condition can be evaluated based on the elevation of nonhigh-density lipoprotein-cholesterol (non-HDL-C), very low-density lipoprotein (VLDL), triglyceride, and apoprotein B levels [8]. The increase in triglyceride levels is due to increased VLDL particle production in the liver and decreased clearance of triglyceride-rich lipoproteins [9]. Thus, it can lead to various advanced complications, such as diabetes mellitus, nonalcohol fatty liver disease (NAFLD), and cardiovascular diseases, such as atherosclerosis [9,10]. Atherosclerosis Cardiovascular Disease (ASCVD) is a fatal and morbid disease [11]. Annually, over 17.9 million individuals (31% of overall deaths) have died due to cardiovascular (CV) diseases worldwide [12].

In previous studies, several synthetic pharmacological approaches have been developed for obesity and dyslipidemia [12]. Several micronutrient compounds from natural products have been reported to be potential agents for improving dyslipidemia and obesity through several mechanisms, including lipase inhibition, appetite reduction, inhibiting lipogenesis, inducing lipolysis, and regulation of lipid metabolism [13]. Butterfly pea (*Clitoria ternatea*/CT) also known as “telang” “teleng” in Java or “celeng” in Bali, Indonesia, is a tropical plant that has been used as a traditional medicine in Indonesia and has been mentioned in Hindu’s Ayurveda [14]. Previously, the *Clitoria ternatea* flower has been investigated and reported to have several potential secondary metabolites for ameliorating obesity and dyslipidemia [15]. According to the Encyclopedia of Herbal Medicines, CT contains 14 types of flavonoid glycosides and 19 types of anthocyanins. The total anthocyanin content in CT is (28.60 ± 0.04) mg/L while 78.75±2.09 mg/g of tannin [15,16]. Anthocyanins and tannins contribute to ameliorating obesity and dyslipidemia through several pathways, including lipase inhibition, lipogenesis reduction, HMG-CoA inhibition that leads to cholesterol synthesis inhibition, lipolysis promotion, increased energy expenditure, and lipid metabolism [13, 17].

But unfortunately, research on CT is still very limited. Therefore, this study aims to investigate the antiobesity and antidyslipidemic effects of *Clitoria ternatea* extract (CTE).

## 2. Materials and methods

### 2.1. Preparation of Clitoria ternatea aqueous extract

CT petals flowers were obtained from several areas in Bangli and Buleleng, Bali, Indonesia. Petal flowers were washed with distilled water and dried for two days, then crushed with a hammer mill crusher (AEG IP54 Lbi 07, Germany) for 30 min. The crushed sample (1 kg) was extracted with 5L of distilled water and boiled for 30 min. The soluble extract was filtered through the nylon. The filtrate was dried by lyophilization. The yield of the aqueous extract of the CT flower was 39.7 g [18].

### 2.2. Determination of antioxidant activity, total anthocyanin, and tannin content

Two sample solutions (KCl buffer with pH 1.0 and ammonia buffer with pH 4.5) were prepared and added with 1 mL of CTE until the volume became 10 mL (dilution factor/DF = 10). Anthocyanin content is indicated by a color change from pink-red to blue-violet while purple-violet color is for tannin content. Then, the absorbance of the sample was measured using a UV-Vis spectrophotometer with a wavelength of 500–700 vis-max and 700 nm. Total anthocyanins are calculated by the formula [19]:


$$\text{Total Anthocyanin}/\text{Tannin}=\frac{\text{Absorbance}\times \text{DF}\times 1000}{55.9\times 1}$$

The diluted of CTE was added into a 5.0 mL volumetric flask, then add 1.0 mL of 0.4 mM 1,1-diphenyl-2-picryl hydrazyl (DPPH) solution as testing specimen and DPPH solution with methanol as control specimen. Then incubated at 37 °C for 30 min. The absorption is read at a wavelength of 515 nm, ten calculated to determine the percentage of inhibition with the formula:


$$\text{Inhibition }(\%)=\frac{\text{Control Absorbance}-\text{Testing Specimen Absorbance}}{\text{Control Absorbance}}$$

### 2.3. Animals study design and feeding

This study has been declared ethically approved with the number 2021.01.1.1228 by The Ethic and Research Unit, Faculty of Medicine, Udayana University. A total of 25 healthy male Wistar rats (8–10 weeks) were prepared and placed at a temperature of 25 ± 1°C, humidity 44%–56%, light and dark cycle for 12 h, and acclimatized for 1 week was done before treatment at the Animal Care Unit, Udayana University. Samples were divided into the following groups:

- ND: Negative control with a normal diet
- HFD: Positive control (High-Fat Diet)
- HFD + CT250: High-Fat Diet + 250 mg/kgBW CTE
- HFD + CT500: High-Fat Diet + 500 mg/kgBW CTE
- HFD + CT750: High-Fat Diet + 750 mg/kgBW CTE

The number of experimental animals in each group was determined by quantification using the following formula by Federer, 1966 [20]:

(t–1) (n–1) ≥ 15
4n ≥ 19
n ≈ 5 per group and total 25 rats

The animals were fed a standard diet of 25 grams per day for the ND group, a high-fat diet (70% standard diet, 20% duck egg yolk, and 10% pork oil) for the HFD and treatment groups, and were given water ad libitum. The HFD group was given HFD for 13 weeks, while the treatment group was given HFD for 10 weeks, followed by HFD + CT for 3 weeks. The extract given would have been previously dissolved to the appropriate concentration.

### 2.4. Weight and obesity index measurement

The weight of the rats was measured at baseline, 5, 10, 11, 12, and 13 weeks using the CAMRY Digital Scale. The Naso-Anal length was measured after the treatment period. These data are used to determine the Body Mass Index and Lee Obesity Index (BMI > 0.68 or LOI > 0.3 is considered obese) [21].


$$\begin{array}{c} \text{BMI}=\frac{\text{Weight }(\text{g})}{{\left[\text{Naso}-\text{Anal Length }(\text{cm})\right]}^{2}}\\ \text{Lee Obesity Index}=\frac{\sqrt[{3}]{\text{Weight}}}{\text{Naso}-\text{Anal Length}}\times 1000 \end{array}$$

### 2.5. Sample collection and organ weight measurement

All animals were anesthetized with 0.5 mL ketamine then cervical dislocation was performed. Blood samples were taken through the eye vein using a microhematocrit and stored in a vacutainer tube. Blood serum was immediately separated by centrifugation and stored at −20 °C. The aorta was collected and weighed then stored in 10% formalin buffer.

### 2.6. Lipid profile analysis

Lipid profile examination using rat blood serum that has been isolated on several parameters, including total cholesterol (TC), triglycerides (TG), and HDL-C according to the instructions from the kit manufacturer (DiaSys Diagnostic Systems GmbH). Indirect calculations were carried out to determine the levels of LDL, VLDL, the Atherogenic Index (AI), and the Cardiac Risk Factors Score through the following equation [22, 23]:


$$\begin{array}{c} \text{VLDL}=\frac{\text{Triglycerides}}{5}\\ \text{LDL}=\text{Total Cholesterol}-\frac{\text{Triglycerides}}{5}\\ \text{Atherogenic Index}=\frac{\text{Total Cholesterol}-\text{HDLC}}{\text{HDLC}}\\ \text{Cardiac Risk Factor}=\frac{\text{Total Cholesterol}}{\text{HDL}} \end{array}$$

### 2.7.Histopathology evaluation

The aorta samples that had been stored in 10% Neutral Formalin Buffer (BNF) for 72 h were dehydrated in 70% and 96% alcohol solutions and then infiltrated with histoplasty paraffin. Slices were performed using a microtome (4 – 5 μm thickness), followed by incubation and staining with Mayer’s hematoxylin-eosin. The preparations were then observed with a light microscope, and the image of the aorta wall was obtained using OptiLab and ObtiLab Viewer 2.2. Images were analyzed using Image J Software.

### 2.8. Statistical analysis

Differences between groups were analyzed using SPSS 25.0 for Windows with a one-way ANOVA test followed by the post-hoc test with an alternative Kruskal-Wallis test followed by the Mann-Whitney test. The p < 0.05 showed a significant difference.

## 3. Results

### 3.1. Phytochemical analysis

Extraction of anthocyanins from CT flowers using the liquid extraction method. The anthocyanin content was obtained at 78.0943 mg/100 g while the tannin content reach. The test for DPPH levels in CTE had an IC50 score of 194.26 μg/mL indicating the weak potency of antioxidant activity [24].

### 3.2. Lipid profile analysis

**Figure 1:**
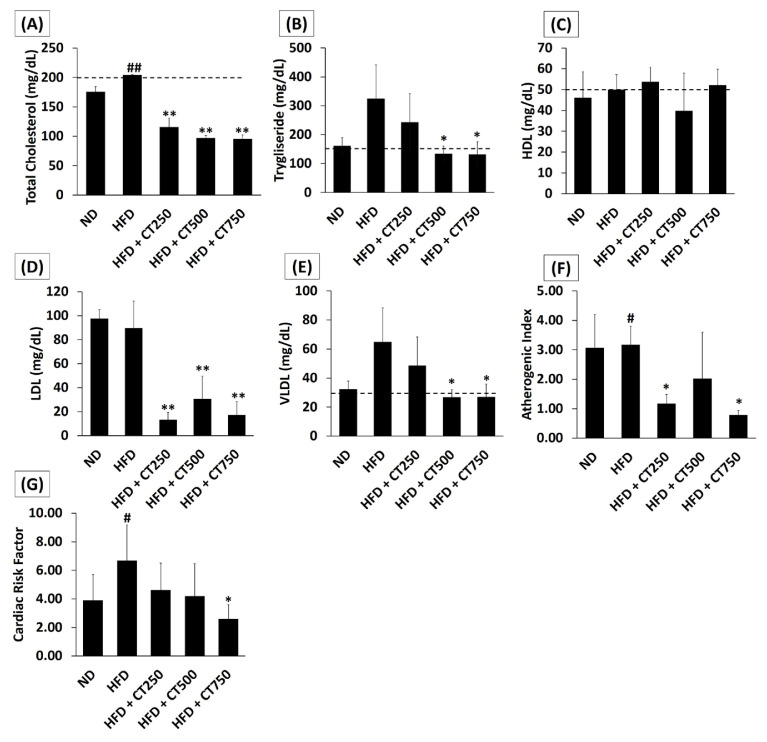
Analysis of lipid profile and the impact on the cardiovascular scoring status. Quantification result on total cholesterol/TC level (mg/dL) (1A). Quantification result on triglyceride/TG level (mg/dL) (1B). Quantification result on HDL level (mg/dL) (1C). Result of indirect measurement of LDL, VLDL, Atherogenic Index, and Cardiac Risk Factor on (1D), (1E), (1F), (1G) respectively. The statistical comparison was carried out among the sample groups using one-way ANOVA and continued with post-hoc or Tamhane T2 test. (* p < 0.05, ** p < 0.05) vs. positive control group. (^#^ p < 0.05, ^##^ p < 0.01) vs. negative control group. TC and TG; normal threshold below (dotted line). HDL; normal threshold above (dotted line).

Significant differences were found in the follow-up analysis (post-hoc), especially in the HFD group and HFD + CT750 (MD 111.12; 95% CI (99.57 to 122.67) mg/dL; p < 0.001) which are represented in Figure 1A. Significant results on postintervention triglyceride differences were obtained in the One-Way ANOVA test (p = 0.002). Figure 1B provides the results of a follow-up analysis of the post-hoc test, which showed the significance, especially between the HFD group and the HFD + CT500 group (MD: 190, 95% CI (92.69 to 287.34) mg/dL, p = 0.001). Figure 1C shows no significant differences between groups in terms of HDL concentration. While Figure 1D–1G shows the potential of CT+750 in reducing LDL, VLDL, atherogenic index, and cardiac risk factor compared to the HFD group (p < 0.05)

### 3.3. Obesity index and body weight analysis

**Figure 2:**
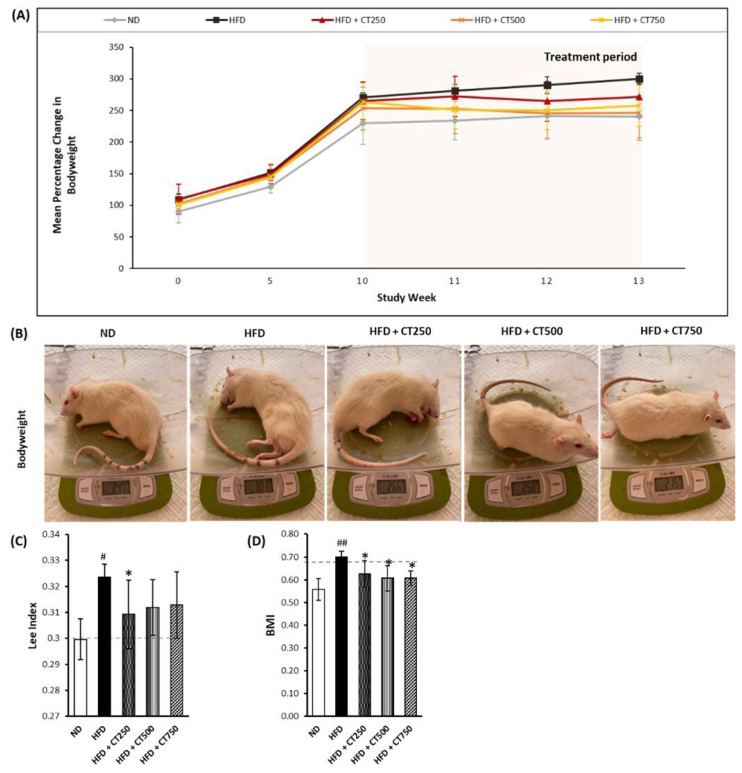
Analysis of obesity index and body weight of Wistar rats. Result of mean percentage change analysis in body weight (2A). Measurement of body weight of sample (2B). Quantification result on Cardiac Risk Index (Lee Index) (2C). Quantification result on Body Mass Index (2D). The statistical comparison was carried out among the sample groups using one-way ANOVA and continued with post-hoc or Tamhane T2 test. (* p < 0.05, ** p < 0.05) vs. positive control group. (^#^ p < 0.05, ^##^ p < 0.01) vs. negative control group.

One-Way ANOVA analysis showed a significant difference in the weight of postintervention rats (p < 0.05). Significant differences were also found in the follow-up analysis (post-hoc), especially in the ND and HFD groups (p = 0.008), HFD and HFD + CT750 group (MD: 56.20, 95% CI (13.89 to 98.51), p = 0.012), and the HFD with HFD + CT750 group (p = 0.020) in Figure 2A. Comparison of rats in each group macroscopically has been reported in Figure 2B. Meanwhile, the Lee obesity index (Figure 2C) and body mass index (Figure 2D) showed significant differences between the HFD group and the CT250 group (p-value of 0.028 and 0.002 respectively).

### 3.4. Peripheral organ weight and microscopic analysis

**Figure 3:**
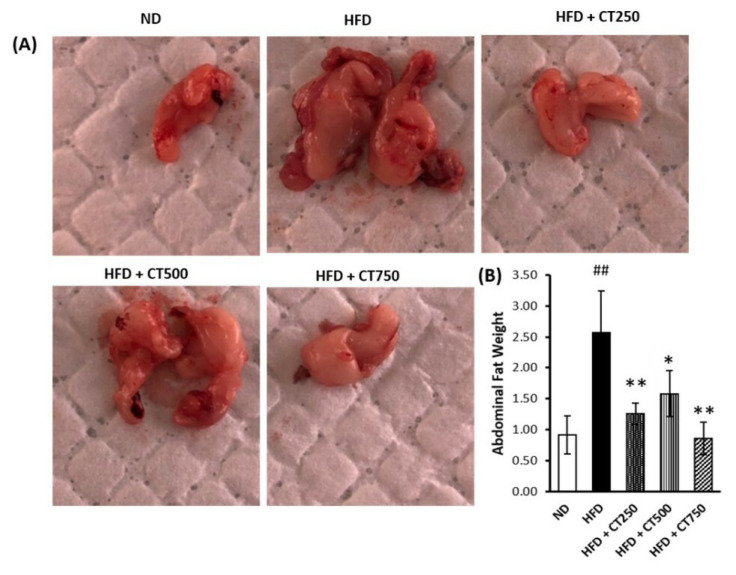
Analysis of peripheral organ weight of Wistar rat. Examination of abdominal fat harvested in the abdominal cavity of sample (3A). Analysis result of abdominal fat weight of the Wistar rat from each group (3B). The statistical comparison was carried out among the sample groups using one-way ANOVA and continued with post-hoc or Tamhane T2 test (* p < 0.05, ** p < 0.05) vs. positive control group (# p < 0.05, ## p < 0.01) vs. negative control group.

**Figure 4:**
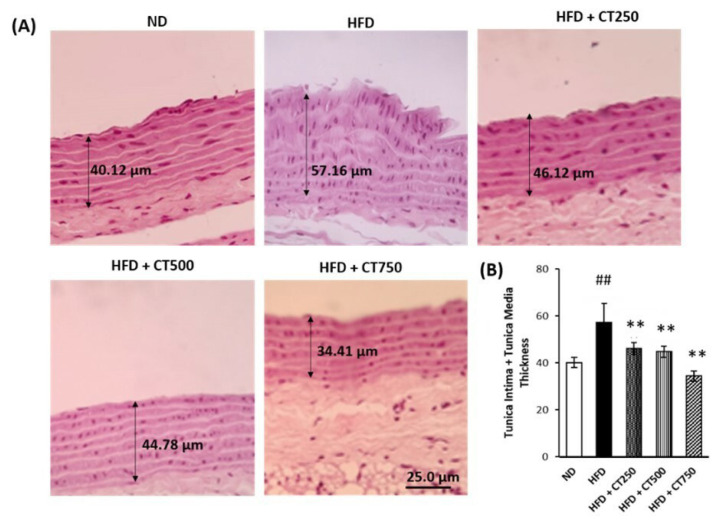
Analysis of microscopic and macroscopic examination on Wistar rat liver and aorta. An examination of tunica intima + tunica media thickness using ImageJ software (4A). The thickness analysis was performed using one-way ANOVA continued by post-hoc. (** p < 0.01) vs. positive control group. (## p < 0.01) vs. negative control group (4B). Stained with H&E 400x magnification.

These results presented in Figure 3A–B indicate that the CTE rich in anthocyanins has an antiobesity effect by reducing the fat weight of experimental animals, especially between the HFD and HFD + CT750 groups (MD: 1.71, 95% CI (1.19 to 2.23), p < 0.001). Microscopic examination of the thoracic aorta can be seen in Figure 4A–4B. Tunica thickening was observed in the negative control group and the treatment group, with an active decrease in thickening seen in the treatment group (57.16 μm vs 46.12 μm; 44.78 μm; 34.41 μm, respectively), especially between HFD and HFD + CT750 group (MD: 22.65, 95% CI (20.11 to 24.4), p < 0.001).

## 4. Discussion

The geographical location of the natural product also influences its antioxidant activity. A previous report conducted by Shafirany et al. [25] showed the IC50 value of CTE is 470 μg/mL, which is better than *Hibiscus sabdariffa L*., or known as rosella, with an IC50 value of 581.5 μg/mL. CT is reported to have antioxidant potential, mainly due to its anthocyanin content [26]. This is supported by the research of Mauludifia et al. [27], which found 85.54 mg/L of anthocyanin in CT at pH 2 as the optimal concentration, and using ethanol extract which gives the best efficacy.

In this study, a decrease in cholesterol levels in the administration of anthocyanin-rich CT extract was observed. This is supported by a systematic review that there was a significant relationship between anthocyanin supplementation and a decrease in total cholesterol observed (MD: −24.06, 95% CI (−45.58 to −2.64) mg/dL., I2 = 93%) [28]. In the cholesterol synthesis mechanism, the mevalonic pathway’s first step is the conversion of 3-hydroxy-3-methylglutaryl-CoA (HMG-CoA) to mevalonic acid by HMGCR. Inhibition of the mevalonate pathway may aid in managing human lipid-associated diseases, which was reported in Wistar rats induced by hyperlipidemia [29]. A decrease in triglyceride levels was found in this study with the administration of CT flower extract. This finding is supported by Thilavech et al. [30] (2021). Compared to the group consuming the high-fat diet (HFD) with the group consuming the HFD + 2 g of dried extract CT plant, postprandial triglycerides began to show a significant decrease at 300 and 360 min after the intervention. CT extract inhibited adipogenesis and reduced triglyceride accumulation levels by downregulating adipogenic gene expression in 3T3-L1 preadipocytes by inhibiting the Akt1 and ERK1/2 signaling pathways, which can prevent adipocyte hyperplasia [31].

This study showed that supplementation of CTE could reduce low-density lipoprotein (LDL) levels because there are flavonoids in CTE. This is supported by a study conducted by Arifah et al. [32] (2022) with the research results reported showing that the treatment group has a significant difference from the normal and negative control groups. Flavonoids work to reduce LDL levels due to the inhibition of HMG-CoA by the active substance simvastatin and reduce hepatic cholesterol production [33]. Through the statistical analysis, a significant decrease in VLDL levels was found in rats, with the results being similar to those found in a systematic review by Ferreira Reis et al. [34], that there was a decrease in VLDL levels in rats and rabbits. Kowalska et al. [35] also said that anthocyanins can inhibit lipid metabolism by down-regulating mRNA lipoprotein lipase (LPL) levels in adipose. With this decrease in LPL activity, VLDL in the body will also decrease [36].

A decrease in the atherogenic index in the administration of anthocyanin-rich in CTE was observed in this study. This is supported by a study [37] that states that there was a relationship between anthocyanin supplementation with a decrease in the atherogenic index which is related to HDL level. The increase in HDL is related to the synthesis of paraoxonase esterase/lactonase 1 (PON-1) to protect lipoproteins and endothelium from oxidation. Weight loss in the administration of anthocyanin from CT extract was observed in this study. This is supported by previous research [38] regarding the effect of giving CT extract in suppressing lipogenesis in the Akt1 and ERK1/2 pathways which leads to suppression of hyperplasia in adipose tissue. According to the results of a meta-analysis by [39] from five studies, it was stated that there was a decrease in BMI in obese patients after anthocyanin supplementation (MD = 0.36, 95% CI = 0.58 to 0.13). The difference was due to the characteristics and control of an HFD when supplementing anthocyanin in the studied subjects [39].

The administration of CT extract in mice with an HFD in this study showed a decrease in BMI. A systematic review conducted by Park, et al. [40] found a significant relationship to a decrease in BMI (95% CI = −0.58 to 0.13; I2 = 0%; p = 0.002). The definite mechanism for reducing BMI is thought to be the process of lipid metabolism and energy expenditure through lipolysis and lipogenesis so that the lipid profile and leptin secretion can be improved [41]. A decrease in the lee index by giving the CT petal extract which contains high anthocyanins was observed in this study. This finding is in line with the research conducted by Yin and Wu [42] which observed administration of high anthocyanin content can also reduce obesity (p < 0.005). This can happen because anthocyanins have been shown to reduce fat levels in the body by inhibiting fatty acid synthesis and increasing fatty acid oxidation [43].The study by Sivamaruthi et al. [44] also found similar results; there was a decrease in fat weight in rats induced by hyperlipidemia after the administration of anthocyanins. Anthocyanins can reduce fat weight a pathway that inhibits pancreatic lipase. The second pathway, anthocyanins can also regulate lipolysis and lipogenesis, thus leading to fat loss. Similarly, the increase in kidney weight was also found in this study significant between HFD and HFD + CT750 group (MD: 0.61, 95% CI (0.25 to 0.96), p = 0.002). A study by Setiawan, et al. [45] with an anthocyanin-rich extract from purple sweet potato showed a similar decrease in tunica intima and media thickness from the diet of atherogenic rats. This reduction was influenced by a decreased inflammatory response from the inhibition of atherosclerotic plaque formation. Macrophages will not accumulate which causes failure of the formation of foam cells that can thicken the walls of the aortic vessels. This theory supports the observable tunica thickening in the negative control group (Figure 4) which did not experience a decrease in thickening compared to the treatment group [45, 46].

Based on these results, the concentration of tannin compounds on CTE obtained the average value is 1424.90 mg/100 g. The different concentrations of Tannins can also be found in *Camellia sinensis L.*, showing the highest tannin-only content of 72.80 ppm [47]. The effect of tannins is antioxidant, radical scavenging, antimicrobial, antiviral, etc. In this study, a decrease in lipid profile levels in the administration of tannin-rich CT extract was observed. This is supported by the research of Sieniawska [48], which found significant results on obese-diabetic rats during the four weeks of supplementation with tannin-cocoa extracts. This can be explained by cholesterol binding to bile acids in the intestine which results in the excretion of cholesterol from the body and inhibit its reabsorption [48].

Another study by Zou et al. [49] found the results of the administration of tannin-persimmon extract significantly (p < 0.05) decreased serum triglycerides, and free fatty acids in rats fed a high-fat diet. The high molecular weight persimmon-tannin (HMWPT) significantly lowers fatty acid synthase (FAS) protein levels and stimulates phosphorylation of AMP-activated protein kinase (AMPK) and down-regulated genes involved in lipogenesis, including sterol transcription factors regulatory element-binding proteins 1 (SREBP1) and acetyl CoA carboxylase (ACC). The different experimental results stated after three doses were used, the tannin extract of *Muntingia calabra L.* was found to have significantly decreased triglycerides, LDL, and HDL (2 weeks, p < 0.005) [49]. Based on the analysis of nonmonotonous doses, in almost all variables a significant improvement in complications from dyslipidemia was found. Improvements were mainly obtained in the HFD+500 and HFD+CT750 groups which showed the best. Similar results were also shown by previous research by Arifah, et al. [32] which reported improvements in Total Cholesterol, LDL, HDL in rats (Rattus Norvegicus) given butterfly pea flower extract at a dose of 400 mg/kgBW.

In conclusion, *Clitoria ternatea* shows antioxidant activity with anthocyanin and tannin content. Furthermore, *Clitoria ternatea* has significant potential effects as an antiobesity and antidyslipidemic agent mediated by its significant effect on reducing lipid profile (cholesterol, LDL, VLDL), body weight, and obesity index (BMI and Lee obesity index). *Clitoria ternatea* also has the antiatherogenic effect which is shown by a significant lowering atherogenic index, and cardiac risk factor index, and also supported by significant differences of tunica layer in the thoracic aorta and abdominal fat accumulation of HFD-induced rats compared to the treatment group with CT.
